# Whole-Body Movement during Videogame Play Distinguishes Youth with Autism from Youth with Typical Development

**DOI:** 10.1038/s41598-019-56362-6

**Published:** 2019-12-27

**Authors:** Adel Ardalan, Amir H. Assadi, Olivia J. Surgent, Brittany G. Travers

**Affiliations:** 1 Zuckerman Mind Brain Behavior Institute, Columbia University, New York, NY USA; 2 Department of Computer Sciences, University of Wisconsin-Madison, Madison, WI USA; 3 Department of Mathematics, University of Wisconsin-Madison, Madison, WI USA; 4 Waisman Center, University of Wisconsin-Madison, Madison, WI USA; 5 Occupational Therapy Program in the Department of Kinesiology, University of Wisconsin-Madison, Madison, WI USA

**Keywords:** Motor control, Human behaviour, Autism spectrum disorders

## Abstract

Individuals with autism spectrum disorder struggle with motor difficulties throughout the life span, and these motor difficulties may affect independent living skills and quality of life. Yet, we know little about how whole-body movement may distinguish individuals with autism spectrum disorder from individuals with typical development. In this study, kinematic and postural sway data were collected during multiple sessions of videogame play in 39 youth with autism spectrum disorder and 23 age-matched youth with typical development (ages 7–17 years). The youth on the autism spectrum exhibited more variability and more entropy in their movements. Machine learning analysis of the youths’ motor patterns distinguished between the autism spectrum and typically developing groups with high aggregate accuracy (up to 89%), with no single region of the body seeming to drive group differences. Moreover, the machine learning results corresponded to individual differences in performance on standardized motor tasks and measures of autism symptom severity. The machine learning algorithm was also sensitive to age, suggesting that motor challenges in autism may be best characterized as a developmental motor delay rather than an autism-distinct motor profile. Overall, these results reveal that whole-body movement is a distinguishing feature in autism spectrum disorder and that movement atypicalities in autism are present across the body.

## Introduction

Autism spectrum disorder (ASD) is a neurodevelopmental disability that affects 1 in 59 children in the US^1^ and has staggering public health costs, estimated at $461 billion annually by 2025^2^. In addition to the primary social communication and repetitive-behavior deficits^3^, individuals with ASD face a diversity of co-occurring motor impairments, including challenges with postural stability^4^, manual motor functions^5–7^, and motor anticipation^8,9^. While motor challenges are not part of the diagnostic criteria for ASD, motor challenges in the first two years of life have been consistently found to be an early indicator of later ASD symptoms and diagnosis^10–14^, and motor challenges in youth with ASD have been found to be associated with core ASD symptoms^6,15,16^.

Motor difficulties in individuals with ASD appear to persist across their life span^7,17,18^ and have been linked to poorer independent-living skills (i.e., dressing, toileting, self-grooming, household tasks, and finances) across multiple age groups^7,19^. For example, manual motor skills were associated with poorer adaptive daily living skills both concurrently and five-to-nine-years later, even after controlling for age and IQ^7^. This link between motor challenges and independent-living skills is important because it suggests that targeting motor skills through intervention could potentially remove barriers to independent living in order to enhance quality of life in ASD.

Yet, we know little about how whole-body movement may be atypical in ASD, with the majority of studies examining just one motor domain at a time. It is important to examine which aspects of movement most robustly differ in ASD compared to typical development (TD), as this information would allow us to detect and treat motor challenges in ASD. A key step toward this goal is to determine which motor domains are the most distinctive in ASD compared to TD. Anzulewicz and colleagues^20^ found that fine motor skills while playing two tablet games were shown to reliably distinguish between 3–6 year-olds with ASD and 3–6 year-olds with TD. Specifically, children with ASD touched the tablet with greater force, had faster screen taps, and had faster, more variable, and larger gestures on the screen. Similarly, Wu and colleagues^21^ found that a quantitative measure of noise during goal-directed reaching reliably distinguished individuals with ASD from individuals with TD. These findings characterize some important aspects of the manual motor profile that most accurately distinguish children with ASD from children with TD. In terms of more gross motor movements, lower-limb movement during walking^22^ and postural control of the head^23^ were found to distinguish between children with ASD and children with TD. While there is emerging evidence regarding how movement of the head, lower limbs, and hands may distinguish ASD from TD, it is unclear how whole-body movements would distinguish youth with ASD and youth with TD and which body parts might drive this effect. Understanding which gross motor features are distinctive in ASD would help elucidate the nature of motor impairments in this population. This is especially true if the motor information can be noninvasively gathered, allowing the participant to freely move and not exacerbating sensory features commonly reported in ASD^24^.

Therefore, the present study had three aims. Using non-invasive, kinematic tracking of body movements during a biofeedback-based balance training in which participants received visual input regarding their body positioning in order to enhance static balance over time^25^, the first aim of the study was to determine whether whole-body movement and postural stability during balance tasks could reliably distinguish between youth with ASD and youth with TD. In other words, could the way that individuals moved during a biofeedback-based balance training^25^ reliably distinguish between the two diagnostic groups? We selected to test this aim with a machine learning (ML) approach rather than traditional null hypothesis testing within each motor domain because of the desire to reduce multiple statistical comparisons, and because of the volume and complexity of the current data set (x,y,z coordinates of 20 joint positions and x-y coordinates of the balance board in multiple training poses across multiple sessions). Based on previous research showing postural stability challenges in ASD^4^ and research showing that fine motor tablet play and goal-directed reaching can distinguish between ASD and TD^20,21^, we hypothesized that a ML algorithm would show strong specificity and sensitivity when classifying the two groups based on all the motor data collected. This aim would determine whether gross motor function could be considered a potential diagnostic marker in youth with ASD.

The second aim was to investigate whether our classification results (i.e., the distance of each individual from the classification boundary) would correspond to key demographic variables (such as age, performance on a standardized motor assessment), and measures of ASD symptom severity. These analyses would allow us to better understand the age-based differences in motor function in ASD and would validate that the classification was driven by motor function and/or ASD symptom severity (and not random noise). Given the distinct developmental trajectory of motor skills in ASD^7^, a key question is whether motor challenges in ASD are more accurately characterized as a developmental delay or as a qualitatively distinct pattern of motor performance. The present data are uniquely poised to answer this question, given the present study’s kinematic data and the age range (7–17 years). For example, if diagnostic groups were reliably distinguished across all ages, this would suggest that whole-body movements are qualitatively distinct in ASD compared to TD. However, if diagnostic groups were only reliably distinguished at certain ages (i.e., all younger participants with TD being classified as ASD), this would suggest that whole-body movements are developmentally delayed in ASD. Examining whether differences in whole-body movements correspond to ASD symptom severity is also an important question. Based on previous research that has shown that social communication and repetitive behavior symptoms in ASD are associated with individual differences in postural stability^6,15,16^, we hypothesized that distance from the classification boundary would predict individual differences in ASD symptom severity. This test would further validate the ML algorithm as a potential tool for clinical use.

The third and final aim was to explore which features of whole-body movement were most informative for a ML algorithm. To date, the majority of studies examining motor skill in ASD have each focused on a specific motor act or region of the body, allowing us to know that at the group level individuals with ASD may struggle with postural stability (see review^4^), grip strength^5,7,26^, and goal-directed reaching^21^. This exploratory aim would determine the specific aspects of whole-body movement that most commonly distinguish between ASD and TD. This aim may shed light on the nature of motor challenges in ASD and might clarify intervention goals related to motor deficits.

To achieve these aims, kinematic and postural sway data were collected in 39 youth with ASD and 23 age-matched youth with TD (ages 7.0–17.9 years), as part of a biofeedback-based videogame training to enhance balance^25^. Table 1 shows the participant demographics. Given the heterogeneity of motor performance reported within the ASD profile^7,27,28^, a 2:1 ratio of ASD to TD participants was selected to allow the motor data to be representative of the diversity within the autism spectrum. An intake session included confirmation of an ASD diagnosis (providing training labels) and a standardized measure of overall motor function outside of the kinematic data^29^.

**Table 1:** Participant demographics.

	ASD (n = 39)	TD (n = 23)	p-value
Male %	95%	74%	—
Age (years), M(SD)	13.08(3.02)	14.09(2.93)	0.20
Age (years), Range	7.80–17.85	7.04–17.69	—
IQ, M(SD)	104.41(14.95)	112.39(9.91)	0.03
IQ, Range	73–136	91–130	—
BOT-2%tile, M(SD)	15.33(14.25)	38.70(20.97)	<0.001
BOT-2%tile, Range	1–62	8–86	—

*Note*: BOT-2 = Bruininks-Oseretsky Test of Motor Proficiency, 2^nd^ edition; IQ = Intelligence Quotient; M = Mean; SD = Standard Deviation.

**Figure 1:**
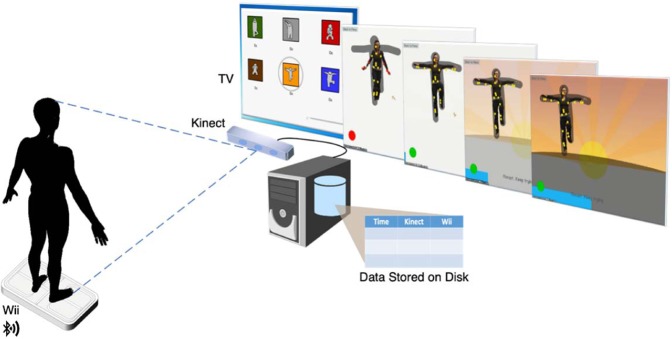
Study setup. Microsoft Kinect camera and Wii Balance Board send the joint kinematic and postural sway data to the computer, which controls the biofeedback-based balance training game and records the data.

Kinematic data from one-hour training sessions were recorded with a Microsoft Kinect Camera, and postural sway data were recorded with a Wii Balance Board (see Fig. 1 for the study set up). Because of the sensory symptoms in ASD^24^, all kinematic data were collected as non-invasively as possible, without markers placed on the body or wires. Because previous work^25^ has demonstrated balance improvements in ASD as a function of this training, training sessions 2–4 (of 18) were used in these analyses to characterize motor skills before the majority of the improvements due to training but after the initial intake assessment (session 1). These data were cleaned, informative features were extracted, and an ensemble of random forest (RF) classifiers were trained. The analysis revealed a sensitive classification of the two groups that was associated with individual differences in age, standardized motor testing, and autism symptom severity. While postural sway and peripheral-limb movements were commonly found to be informative for the classification, the results suggested that no single motor domain was able to reliably distinguish between the two groups. In other words, combined information about movement across the body was necessary for the accurate classification.

## Results

### Classification results

Table 2 shows the overall results of our stratified 5-fold cross-validation (CV) analysis, as well as the results for individual folds. We chose 5-fold instead of the more commonly-used 10-fold CV to make each individual fold contain a reasonable number of participants in both groups. We obtained a mean precision of 0.75, mean recall of 1.00, mean specificity of 0.43, mean Matthew’s Correlation Coefficient^30^ (MCC) of 0.56, and mean F1 score of 0.86 (see Supplementary Methods for definitions). Hence, our approach was highly accurate in using gross motor information to distinguish between the ASD and TD groups. The variability of the fold results appears to be due to the heterogeneity within the ASD profile, which is further explored below in the individual difference results.

**Table 2:** Accuracy of our classification results for stratified 5-fold cross validation. Results for individual folds as well as the average accuracy measures over all folds are reported.

	Precision	Recall	Specificity	MCC	F1
Fold 1	0.80 (8/10)	1.00 (8/8)	0.60 (3/5)	0.69	0.89
Fold 2	0.73 (8/11)	1.00 (8/8)	0.40 (2/5)	0.54	0.84
Fold 3	0.73 (8/11)	1.00 (8/8)	0.40 (2/5)	0.54	0.84
Fold 4	0.80 (8/10)	1.00 (8/8)	0.50 (2/4)	0.63	0.89
Fold 5	0.70 (7/10)	1.00 (7/7)	0.25 (1/4)	0.42	0.82
Average	0.75	1.00	0.43	0.56	0.86

### Differential experiments for classification results

Because the current project used only a single Kinect Camera for data collection, we were concerned that the z-dimension would be less accurate than the x- and y-dimensions. Therefore, we performed a follow-up analysis in which we trained an ensemble of random forest classifiers on the x-y projection of the body markers, where all z-direction data were dropped. With the z-direction data removed, we obtained a mean precision of 0.75, mean recall of 0.95, mean specificity of 0.44, mean MCC of 0.48, and mean F1 score of 0.83. The similarity of the classification results with and without the z-dimension suggest that the original results are not driven by the potentially less-accurate characterization of the z-dimension with the Kinect camera.

To investigate the effect of oversampling the ASD subpopulation, we performed a follow-up balanced classification with 23 participants in each group. The 23 participants in the ASD group for this analysis were randomly selected among the 39 participants with ASD. With these equally sized groups, we obtained a mean precision of 0.81, mean recall of 0.79, mean specificity of 0.76, mean MCC of 0.56, and mean F1 score of 0.79. Both analyses had identical MCC, but the balanced analysis had slightly lower recall. This suggests that because of the high variability in motor performance in the ASD population, the classification model trained with a smaller ASD sample was less able to identify all of the ASD participants, which supports the decision to oversample the ASD population in this study.

### Individual difference results

**Figure 2:**
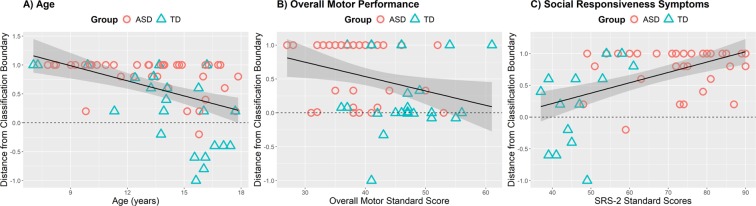
Correlation analysis of the distance from classification boundary vs. (**A**) age, (**B**) overall motor performance and (**C**) social responsiveness symptoms.

The distance of each individual from the classification boundary (calculated as ASD classification probability (a continuous measure), rescaled to the [−1, +1] interval) was significantly correlated with age, *r*(60) = −0.42, *p* < 0.001, standardized motor scores^29^, *r*(60) = −0.42, *p* < 0.001, standardized balance scores^29^, *r*(60) = −0.30, *p* = 0.02, autism symptom severity scores^31^, *r*(52) = + 0.49, *p* < 0.001, and repetitive behavior/restricted interest scores^32,33^, *r*(52) =  + 0.47, *p* < 0.001, using a Pearson correlation coefficient. Figure 2 shows that younger participants, participants with poorer motor skills, and participants with more severe autism and autism-like symptoms were more likely to be classified in the ASD group by the ML algorithm. Because of the potential non-linearity of the classification boundary measure, we performed Spearman correlations and found the exact same pattern of results. Similarly, because of potential ceiling and floor effects of our classification boundary measure, we additionally performed tobit models^34^ and found the exact same pattern of results, suggesting that ceiling and floor effects were not detrimental to the correlational analyses reported above. Because the groups differed in full scale IQ and sex, we performed a follow-up regression analysis examining the distance from the classification boundary as a function of age, motor skills, autism symptom severity scores, IQ, and sex. In this analysis (see Supplementary Table S1), IQ and sex were not significant predictors and did not improve the statistical model according to the adjusted *R*^2^, suggesting that group differences in IQ and sex were not important predictors of our classification results. Therefore, each participant’s distance from the classification boundary was found to be associated with outside measures of motor skills and autism symptom severity, suggesting that the ML outcome was sensitive to both motor skills and autism symptoms and unlikely to be due to random noise. Further, the classification results were not sensitive to other measures like IQ and sex, although the classification results did appear to be sensitive to age.

### Key features results

**Figure 3:**
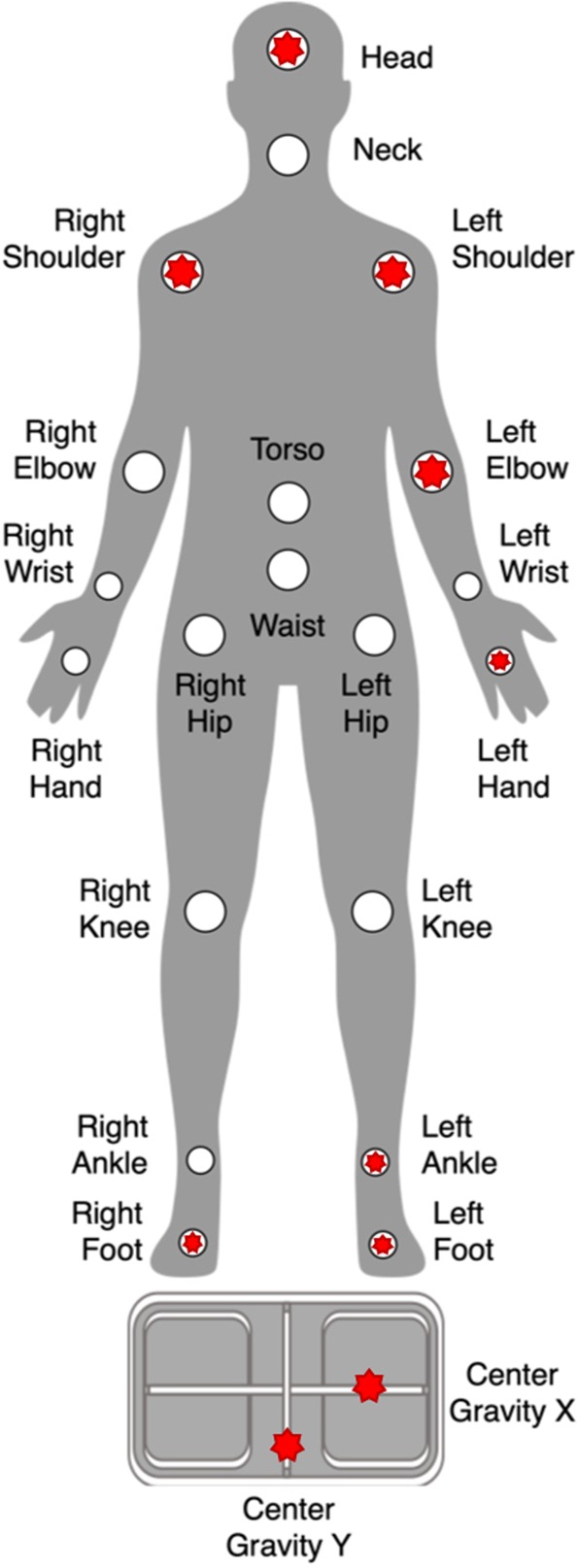
Top-ranked body features used by RF classifiers, marked as red stars.

**Figure 4:**
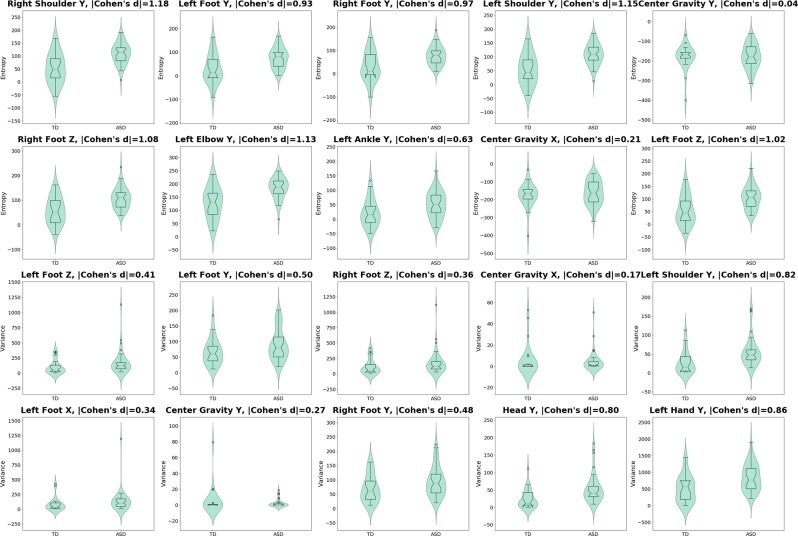
Distributions of top-ranked feature values for ASD vs. TD participants, and Cohen’s d effect sizes of their differences.

To determine the most informative features for our classification algorithm, we ranked the features using total mean decrease in impurity (MDI) measure^35^. Table 3 shows the top 20 features and their MDI scores, and Fig. 3 illustrates the body parts associated with the top-ranked features. To check whether the important features are consistent across various folds, we created a top-20 feature list for each fold (using the corresponding RF ensemble) and then calculated the overlap with the overall top-20 feature list from Table 3. The average overlap is 0.84, indicating that our RF ensembles were highly consistent. Figure 4 shows the violin plots of these features along with Cohen’s d effect sizes^36^ (the assumption of Gaussian-like distributions of the features for each group can be validated by visually investigating the violin plots). The results demonstrate that the ASD group tended to have higher variability and higher entropy in their kinematic movements. Half (10 of 20) of the effect sizes for group differences in the top 20 features were large (Cohen’s *d* ≤ 0.80); i.e., head, right and left shoulders, right and left feet, left elbow, and left hand. However, almost all the other half (8 of 20) of group differences were small effect sizes (Cohen’s *d* ≤ 0.50), suggesting that the success of the ML algorithm was due to the aggregate of these features rather than driven by any single feature.

**Table 3:** Top-ranked features used by RF classifiers, using MDI scores.

Feature Name	MDI
Right Shoulder Y (entropy)	0.89
Left Foot Y (entropy)	0.80
Left Foot Z (variance)	0.80
Right Foot Y (entropy)	0.72
Left Foot Y (variance)	0.72
Right Foot Z (variance)	0.71
Left Shoulder Y (entropy)	0.68
Center of Gravity Postural Sway Y (entropy)	0.66
Right Foot Z (entropy)	0.63
Center of Gravity Postural Sway X (variance)	0.62
Left Shoulder Y (variance)	0.62
Left Foot X (variance)	0.61
Left Elbow Y (entropy)	0.60
Center of Gravity Postural Sway Y (variance)	0.59
Left Ankle Y (entropy)	0.57
Center of Gravity Postural Sway X (entropy)	0.57
Right Foot Y (variance)	0.56
Head Y (variance)	0.55
Left Hand Y (variance)	0.55
Left Foot Z (entropy)	0.55

## Discussion

Whole-body movement during a balance task was able to distinguish between youth with ASD and youth with TD. Therefore, in addition to specific aspects of fine-motor and gross-motor performance that has been found to distinguish between the diagnostic groups^20–23^, whole-body gross motor features also appear to be a distinctive feature between diagnostic groups. Both fine and gross motor impairments have been observed in ASD^37^, and whole-body assessments would likely lead to a better understanding of the motor challenges at both the individual and population levels. Importantly, gross motor features in the present study were unobtrusively recorded without attached wires or markers and during videogame play, offering an ecologically valid method of quantifying gross motor movement in this population.

The results further found that the classification based on whole-body kinematic data was sensitive to individual differences in overall motor skill, balance skill, social responsiveness symptom severity, and repetitive behavior symptom severity. These relations validate the classification approach, showing that the classification results aligned with individual differences in motor challenges and autism symptom severity and were unlikely to be due to random noise. In other words, the non-invasive whole-body tracking during videogame play highly corresponded to the results of the lengthy motor and autism diagnostic assessments. Further, the sensitivity of the classification model to individual differences in motor and autism symptom severity also helps explain the variability in model accuracy across different CV folds, suggesting that which individuals were selected to train the classification model led to differing degrees of classification accuracy. While it is a promising first step to be able to use motor data to distinguish between a heterogeneous group of youth with ASD and youth with TD, clinical applications will require that this approach be able to distinguish ASD from children who are referred for an ASD diagnosis but likely have non-ASD neurodevelopmental conditions. Therefore, future research will be needed to determine if classification using whole-body kinematics can be applied to a population of children who are referred for an ASD diagnosis, distinguishing between those who go on to receive a diagnosis and those who do not.

The classification results were also sensitive to age. Previous studies using motor information to distinguish ASD from TD have similarly used age ranges during which substantial motor development is occurring (16–31 months^23^, 3–6 years old^20^; 4–12 years old^22^, and 7–30 years old^21^). However, a unique aspect of our study is that we explicitly examined the role of age effects in the classification algorithm. Specifically, all but one of the participants with TD under the age of 15 were misclassified by the algorithm to the ASD group. Therefore, younger participants with TD were interpreted to have similar motor patterns as those with ASD. From the literature, it has been unclear whether motor patterns are qualitatively distinct in ASD compared to TD or whether motor patterns are representative of a delay. Most of the youngest participants with TD being misclassified into the ASD group suggests that gross motor patterns in ASD are more consistent with a motor delay, which is important for conceptualizing how gross motor challenges are treated in this population. Further, this age-sensitivity suggests that this gross-motor classification may be most successful at distinguishing ASD from TD in older adolescents, consistent with previous findings of a growing difference in motor skills between ASD and TD with age^7,17,26^. As most ASD diagnoses take place in early childhood, the present age findings suggest that this tool may have limited utility as a diagnostic assessment for ASD early in life. However, the more reliable classification in the older age groups combined with the link between the classification output and overall motor ability suggest that this tool could potentially be used as a screener for adolescents with ASD who may need physical and occupational therapies to address motor challenges, a possibility to be examined in future research. Further, as there is an aging population of individuals with ASD, future research is needed to examine whether this non-invasive method could be used beyond adolescence to reliably determine which older adults with ASD may be at a higher risk for falls.

An exploratory goal of the present study was to determine if specific features of movement captured by our algorithm were most indicative of diagnostic status. Of the features used by RF decisions trees, we found that postural sway, feet, head, and shoulder movements were the most commonly used features to determine group status in the ML algorithm, with the ASD group demonstrating more variability and entropy in movement. However, of those features, only movement of the head, shoulders, feet, left elbow, and left hand had large effect sizes when distinguishing between the two groups. Therefore, it is unlikely that any one movement feature could be considered the most indicative of ASD, and it is more likely that all the movement features must be taken into account in order to make the most accurate classification between ASD and TD. In other words, a unique finding from the present study is that small-to-medium movement differences between ASD and TD were present across the body, suggesting that motor challenges are likely broadly related to the central nervous system rather than localized to specific areas of the body.

The notable limitations of this work include the relatively small sample size (particularly in the TD group), a limitation which we tried to offset by the number of repeated measures for each individual. Additionally, these data were collected during a balance training, in which participants were asked to hold as still as possible. Understanding motor features that distinguish ASD and TD groups in more dynamic movement tasks was not explored but will be a key avenue for future investigations. In the analysis of more dynamic tasks, joint configuration space and end effector space will likely be important variables for classification, although in the present static standing tasks, we considered any movement (regardless of the relationship among the joints) to be an important variable to measure. Further, we employed a more traditional ML model (ensemble of RF classifiers) so that we could examine the underlying features leading to the diagnostic classification. However, since no one feature of movement seemed to be particularly indicative of ASD versus TD, it is likely that more sophisticated methods, such as deep learning, might be more accurate for detecting group differences. In particular, it will be important for future research to understand if similar classification results can be achieved after just one session, as this would best mimic how this task could be used in the clinical environment. Further, given the motor heterogeneity in ASD, a key next step in a larger sample will be to determine whether these motor data render meaningful clusters or subgroups within the ASD group.

In all, strong diagnostic classification between youth with ASD and youth with TD was rendered by a non-invasive, markerless tracking of whole-body movements and postural sway during a biofeedback-based balance training video game. Not only were the present gross motor measures able to distinguish participants at the group level, but the measures were found to correspond to individual differences in motor skill, balance skill, age, and autism symptom severity. Further, the classification results suggested generally higher variability and higher entropy across movements in the ASD group, although no single bodily movement seemed to reliably distinguish between the groups. Overall these results suggest that atypicalities in gross motor movements in ASD are likely persistent across the body and likely representative of a developmental motor delay rather than a distinct motor profile.

## Method

### Participants

The study was approved by the University of Wisconsin-Madison Institutional Review Board, and all methods were carried out in accordance with the ethical standards of the institutional research committee and with the 1964 Helsinki declaration and its later amendments or comparable ethical standards. All parents provided written informed consent, and all youth provided informed assent. Thirty-nine youth with ASD (ages 7.80–17.85 years, *M* = 13.57, 3 females) and 23 youth with TD (ages 7.04–17.69 years, *M* = 13.88, 6 females) participated in this study. All participants were recruited through fliers in the community, the Waisman Center registry database, and word of mouth. The participants with ASD had a prior clinical diagnosis of ASD that was supported by meeting criteria for ASD on Modules 3 or 4 of the Autism Diagnostic Observation Scale, 2nd edition (ADOS-2)^38^ or by meeting cutoffs on the Social Communication Questionnaire (SCQ)^39^ and the Social Responsiveness Scale, Revised (SRS-2)^31^. In addition, participant’s severity of repetitive behaviors and/or restricted interests was measured with the Repetitive Behavior Scale-Revised (RBS-R)^32,33^. Participants were required to not have tuberous sclerosis, fragile X, a history of severe head injury, hypoxia ischemia, or intellectual disability (confirmed via IQ testing using the Wechsler Abbreviated Scale of Intelligence, Second Edition^40^). The groups were matched on age, *t*(60) = 1.29, *p* = 0.20, and performance IQ, *t*(60) = 1.51, *p* = 0.14, but the groups were marginally different in full scale IQ, *t*(60) = 2.28, *p* = 0.03. Pre-training motor and balance assessments were performed using the Bruininks-Oseretsky Test of Motor Proficiency, Second Edition^29^.

### Materials

The biofeedback-based balance training set up and protocol have been previously detailed^25^. In brief, the study started with an intake session, in which participants completed standardized IQ and motor assessments. The group with ASD additionally completed autism diagnostic measures. After intake, 60-minute video game training sessions took place three times per week for six consecutive weeks. Each training session included training on a biofeedback-based videogame we developed to enhance balance in youth with ASD. Within this game, participants held 10 static poses inspired by Yoga and Tai Chi practices: karate kid (i.e., crane) (left foot and right foot), tree pose (left foot and right foot), arm-to-knee (left foot and right foot), standing side bend (left side and right side), energy ball, and hug the tree (see Supplementary Fig. S1).

The biofeedback-based balance training game’s software integrated a Microsoft Kinect Camera and a Nintendo Wii Balance Board to monitor the participant’s whole-body kinematic movements and postural sway. Participants stood on the Balance Board in front of a 51″ wall-mounted television. The participant saw him or herself on a blank television screen with joint dots projected on the participant’s image to represent joint coordinates. A research assistant manually adjusted a shadow of the to-be-copied pose to the size of the participant. When the participant’s body was positioned within the shadow, all joint dots turned yellow. However, any joint dot not within the shadow turned red to alert the participant of the error. Participants were encouraged to hold each pose as long as possible. Delimited text files containing the force sensor data from the Balance Board and the x,y,z coordinates of the joint dots from the Kinect Camera were recorded during each pose. To additionally validate these values, we video recorded all sessions with a Snagit screen capture and a GoPro video camera (directed at participant).

### Data acquisition and pre-processing

In all, 8,350 data files were collected from 62 participants. Each file represented the timeseries of a participant performing one of 10 balance poses. We then restricted our dataset to training sessions 2–4, which amounted to 1,860 data files. Each data file had 62 dimensions that varied as a function of time: (1) 3D positions of 20 different joint dots on participant’s body captured by the Kinect Camera, and (2) 2D position of the participant’s center of gravity on the Wii Balance Board (Fig. 1).

Because markerless kinematics have the potential to produce noisy measurements, we checked the Kinect Camera data against Snagit and GoPro videos in a small subset of the data. To address very rare missing values in the timeseries (<0.000001%), we propagated the last valid value of that feature forward. Further, we noticed that there were often high-frequency components of the movement that would be impossible for a human to produce and that were not present in non-Kinect videos of the movements. To remove this noise, we applied a multi-level discrete wavelet transform (using Haar wavelet) to each dimension and discarded the high-frequency components (>level 5, determined by investigating the high frequency movement a small subset of the videos). Another source of noise was that the game was designed to record both before and after completion of the pose (to err on the side of over-including movement data), but this meant that non-balance movements were included at the beginning and end of the data file. To discard the non-balance parts of the timeseries, we took a maximum of 10,000 frames (based on length of balance pose within each video) from the middle of the timeseries and trimmed surrounding data (videos shorter than 10,000 frames were not trimmed). This procedure trimmed ~26% of data from each file on average. We manually checked these trimmings in a small subset of files and confirmed that the retained data represented performance of the task. Finally, to detect and discard an entire timeseries with extreme variance (representative of computer or camera malfunction, poses where the computer had started but the participant did not do the pose, or poses where the Kinect Camera recorded other features than the participant’s movement), we calculated the variance of all dimensions within all data files for a specific pose. We fit an exponential distribution to the variances of each dimension and discarded data files corresponding to each pose that had extreme outliers (i.e., variances in the 10^–7^ tail of that distribution). This cleaning approach dropped 5.2% of the data files (5.3% in ASD and 5.0% in TD). Separately, blinded human coders examined a small subset of the data using the graphs of the kinematics and the non-Kinect video recordings to identify files that were not representative of the participant completing the pose. The automatically discarded data files corresponded to the non-representative files identified by the manual cleaning procedures. We emphasize that after spending manual effort in creating and tuning the data cleaning pipeline described above, this pipeline runs completely automatically for the rest of the data and any new data to be processed using this pipeline in the future.

### Feature extraction and selection

We used a fixed sliding window (size = 20 and shifting value = 10) to extract features from each cleaned timeseries (a window of size 20 corresponded to ~10 seconds of gameplay after applying the wavelet transform). We started at the beginning of cleaned timeseries and calculated the entropy (uncertainty) and variance (spread) of each of the 62 dimensions for each window, creating a feature vector of length 124 (2 × 62). We then slid the window and repeated the above process to convert each timeseries to a collection of feature vectors, which were subsequently used to train and test the classification model. The number of feature vectors generated from each timeseries was different since the lengths of the timeseries corresponding to various sessions were different (see Supplementary Table S4 for more details). Data cleaning and feature extraction were performed on all data files from training sessions 2–4.

### Machine learning data analysis

**Figure 5:**
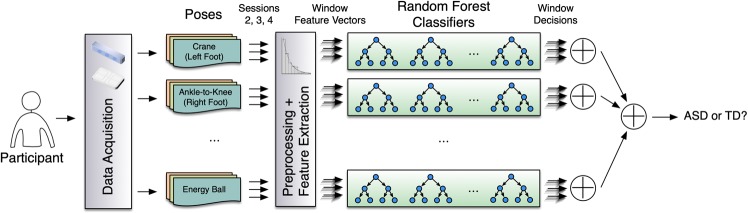
Architecture of our data acquisition and analysis pipeline.

Figure 5 shows the architecture of our classifier ensemble in the context of the end-to-end pipeline. We used an ensemble of 10 RF classifiers^41^ where each RF dealt with the input vectors from a specific pose. Each RF in turn consisted of 10 decision trees (DTs) trained using the CART algorithm^35^ (see Supplementary Methods for training parameter settings) and the decisions made by individual DTs were combined by averaging the scores of all the DTs. We used the following approach to classify each participant as ASD or TD. First, we fed all the feature vectors corresponding to the participant performing a specific pose, into the RF corresponding to that pose and used majority voting to make a partial decision based on the evidence we had for the current pose. After calculating all of the partial decisions for various poses, we again used majority voting to make a final decision to classify the participant as ASD or TD.

We chose RF as our classification model, based on the results of our model selection analysis (see Supplementary Table S2), our prior experience, and the interpretability requirement for our solution. While other classification models are interpretable to various degrees, RFs are very clear in identifying import features and are shown to have high accuracy in a wide range of problems^42,43^.

To evaluate our classification approach in Aim 1, we used stratified 5-fold CV to divide participants into five groups, and then reported the average CV accuracy measures as well as individual fold results. The accuracy measures we evaluated were precision, recall, specificity, MCC and F1 score (see Supplementary Methods for definitions).

To evaluate demographic correlates of classification output in Aim 2, we calculated Pearson R correlations examining each individual’s distance from the classification line (ranging from −1 (TD classified) to +1 (ASD classified) with 0 = boundary line) as a correlate of age, standardized motor score and balance score from the BOT-2, and ASD symptom severity (SRS-2 and RBS-R scores). Because of anticipated floor-level symptoms on the SRS-2 and RBS-R, the first eight participants with TD were not administered these measures. However, procedures were amended to collect SRS-2 and RBS-R data from all participants.

To determine the motor features that were most informative in distinguishing between the two groups (Aim 3), we calculated the total MDI score for each feature as the sum of its normalized MDI scores across all 10 RF classifiers. We then used these scores to rank the features in descending order and reported the top-ranked features.

## Supplementary information

Supplementary information 

## Data Availability

The datasets analyzed during the current study are available from the corresponding author upon reasonable request.
