# Supplementary material for: Whole-Body Movement during Videogame Play Distinguishes Youth with Autism from Youth with Typical Development
Source: Sci Rep. 2019 Dec 27;9:20094. doi: 10.1038/s41598-019-56362-6 (PMC6934713; doi:10.1038/s41598-019-56362-6)
Supplement: Supplementary file 1 — Supplementary information [file 41598_2019_56362_MOESM1_ESM.pdf]

Whole-Body Movement during Videogame Play Distinguishes Youth with Autism from Youth with Typical Development

Adel Ardalan[1,2], Amir Assidi[3], Olivia J. Surgent[4], & Brittany G. Travers[4,5,*]

[1]Zuckerman Mind Brain Behavior Institute, Columbia University, New York, NY USA

[2] Department of Computer Sciences, University of Wisconsin-Madison, Madison, WI USA

[3]Math Department, University of Wisconsin-Madison, Madison, WI USA

[4]Waisman Center, University of Wisconsin-Madison, Madison, WI USA

[5]Occupational Therapy Program in the Department of Kinesiology, University of Wisconsin-Madison, Madison, WI USA

*Corresponding author: Brittany G. Travers, 1500 Highland Avenue, Room 435, Madison, WI 53705, btravers@wisc.edu, (608) 263-0282

Supplementary Information

Supplementary Methods

Accuracy measures. To choose a classification approach, we conducted model selection analysis using various models, namely random forests (RF), support vector machines (SVM) with linear and radial basis function (RBF) kernels, k-nearest neighborhood (KNN) and logistic regression (LR). We plugged each of the above models into our classification architecture (see Figure 5 in the Method section) to build our end-to-end classifier. To evaluate these approaches, we used precision, recall, specificity, Matthew's Correlation Coefficient (MCC) and F1 score, defined as

$$\mathrm{Precision} = \frac{\mathrm{TP}}{\mathrm{TP} + \mathrm{FN}}$$

$$\mathrm{Recall} = \frac{\mathrm{TP}}{\mathrm{TP} + \mathrm{FP}}$$

$$\mathrm{Specificity} = \frac{\mathrm{TN}}{\mathrm{TN} + \mathrm{FP}}$$

$$\mathrm{MCC} = \frac{\mathrm{TP} \times \mathrm{TN} - \mathrm{FP} \times \mathrm{FN}}{\sqrt{(\mathrm{TP} + \mathrm{FP})(\mathrm{TP} + \mathrm{FN})(\mathrm{TN} + \mathrm{FP})(\mathrm{TN} + \mathrm{FN})}}$$

$$\mathrm{F1\ Score} = \frac{2 \times \mathrm{Precision} \times \mathrm{Recall}}{\mathrm{Precision} + \mathrm{Recall}}$$

where TP, TN, FP and FN are True Positives, True Negative, False Positives and False Negatives, respectively.

RF training parameter settings. For training our RF classifiers, we used maximum DT depth of 13, the Gini split criterion, 10 DTs per RF, bootstrapping, and balanced class weights. We chose these parameters through grid search, while trying to prevent overfitting.

Using principal component analysis. We report a baseline analysis below which is commonly used to benchmark machine learning pipelines. First, we performed principal component analysis (PCA) on our data and retained the first 16 principal components which explain >98% of the variance. Hence, our data was projected onto a space with lower dimensionality compared to the original feature space. We then trained and evaluated our classification ensemble on this low-dimensional data using all the base classifiers used in our study and stratified 5-fold CV. The results, reported in Supplementary Table S3, were inferior to our main results using the original feature space and RF ensemble. We posit that this is, at least in part, a result of PCA (which is an unsupervised dimensionality reduction method) ignoring the label information available to the RF ensemble.

To assess whether PCA can effectively identify important features from the original feature space, we sorted the features by the mean of absolute values of their coefficients in the principal components. The top-20 features have only 25% overlap with the top-20 features based on our RF classifier ensembles trained/evaluated in the original feature space, ranked using MDI score, which in turn supports our intuition about the reduction in performance when reducing dimensionality while ignoring the labels.

Supplementary Tables

Supplementary Table S1. Summary of unstandardized beta coefficients from multiple regression analysis examining predictors of distance from the classification boundary (0=boundary, >0 = autism spectrum disorder [ASD], <0 = typical development [TD]). Significant effects are bolded. The overall model was significant, $F(5,48) = 7.23$, $p < .001$, adjusted $R^2 = .37$. IQ and sex were not significant predictors nor did IQ and sex improve the statistical model according to AIC (Akaike, 1974). This finding suggests that group differences in IQ and sex were not important predictors of our classification results.

	b	SE	*t*-value	*p*-value
Intercept	1.41	0.71	2.00	.05
Standardized Motor Score	-0.02	0.01	-1.55	.12
**Age**	**-0.05**	**0.02**	**-2.46**	**.02**
**Autism Symptom Severity (SRS-2 total standard score)**	**0.01**	**0.004**	**2.81**	**.007**
IQ	0.0004	0.005	0.08	.93
Sex	-0.14	0.18	-0.77	.45

Supplementary Table S2. Preliminary analysis results to choose a classification approach. The accuracy numbers are averages from stratified 5-fold cross validation. Random forest model outperforms other popular models evaluated here.

	Average Precision	Average Recall	Average Specificity	Average MCC	Average F1
**RF**	**0.75**	**1.00**	**0.43**	**0.56**	**0.86**
SVM (Linear)	0.79	0.82	0.60	0.48	0.80
SVM (RBF)	0.63	1.00	0.00	0.00	0.77
KNN	0.75	0.95	0.44	0.48	0.83
LR	0.83	0.82	0.65	0.53	0.80

Supplementary Table S3. PCA analysis results. The accuracy numbers are averages from stratified 5-fold cross validation.

	Average Precision	Average Recall	Average Specificity	Average MCC	Average F1
RF	0.74	0.95	0.40	0.44	0.82
SVM (Linear)	0.90	0.67	0.82	0.52	0.73
SVM (RBF)	0.71	0.93	0.34	0.36	0.80
KNN	0.76	0.95	0.48	0.52	0.84
LR	0.87	0.67	0.78	0.48	0.71

Supplementary Table S4. Statistics of number of feature vectors extracted from analyzed timeseries using the sliding window approach.

	Min.	Max.	Mean	Variance
ASD	201	2144	1765.28	238189.35
TD	603	2144	2010.10	72310.58
Overall	201	2144	1856.24	190556.20

Supplementary Figures

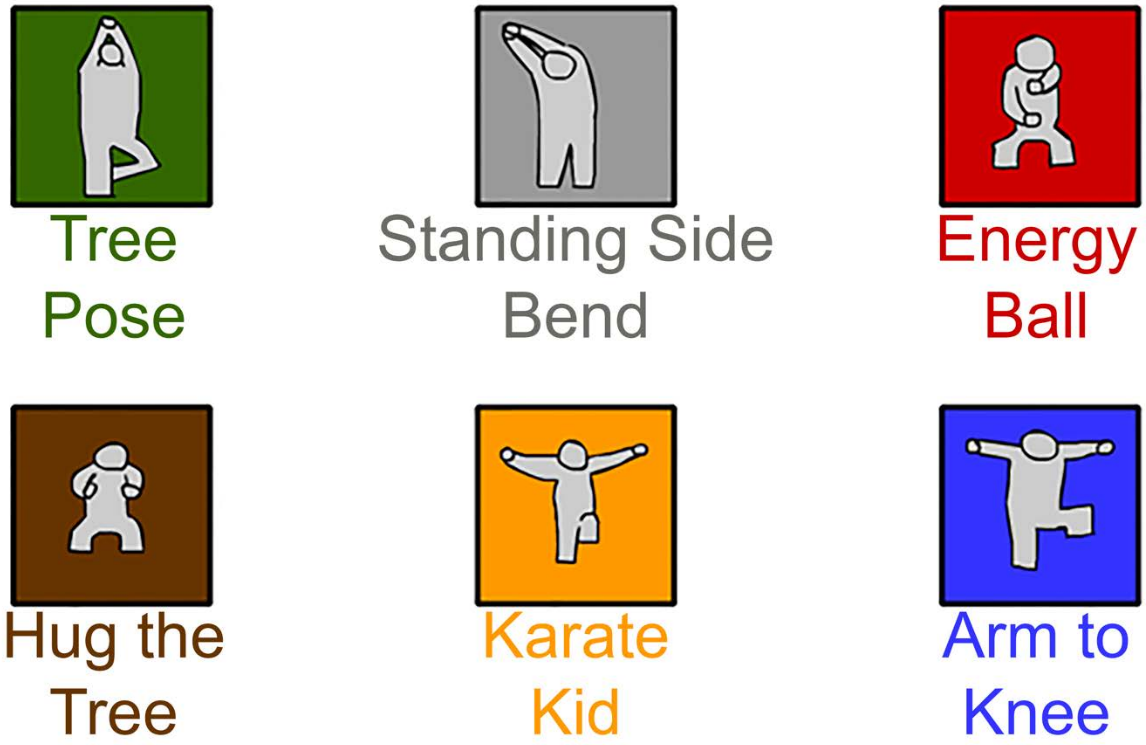

Supplementary Figure S1. Balance training poses used in our video game setup.
